# Multiple Origins of Neurons From Secretory Cells

**DOI:** 10.3389/fcell.2021.669087

**Published:** 2021-07-07

**Authors:** Leonid L. Moroz

**Affiliations:** ^1^Department of Neuroscience, McKnight Brain Institute, University of Florida, Gainesville, FL, United States; ^2^Whitney Laboratory for Marine Biosciences, University of Florida, St. Augustine, FL, United States

**Keywords:** evolution of nervous system, neurotransmitters, Ctenophora, Placozoa, Cnidaria, Porifera, scRNA-seq, behavior

## Why Are Neurons Different? Introduction

The elusive diversity of neurons puzzled neuroscientists since discovering the first nerve cells in the 1830s. Quantitative information about neuronal diversity began to flow from the middle of the twentieth century. At that time, microelectrode and histochemical tools were applied to vertebrate and invertebrate preparations. Simpler nervous systems of some gastropod mollusks, annelids, and nematodes revealed identified neurons with defined transmitter specificity and functions. Early systematic studies pointed out that most of the neurons composing their nervous systems might be unique (Bullock and Horridge, 1965). That revelation provided tractable experimental preparations to decipher cellular bases of behaviors (Kandel, 1976, 2001; Kuffler and Nicholls, 1976).

Today, with advances in single-cell (epi)genomics and transcriptomics, the astonishing diversity of neuronal cell types exceeds any imagination (Moroz, 2018). *The most straightforward question is, how different are the neurons? But more fundamental questions are: Why are neurons different? Why are there so many neurotransmitters? Why are neurotransmitters different?* These questions have been addressed by many (Kandel, 1979; Van Vallen, 1982; Bloom, 1984), aiming for functional aspects.

**Figure 1:**
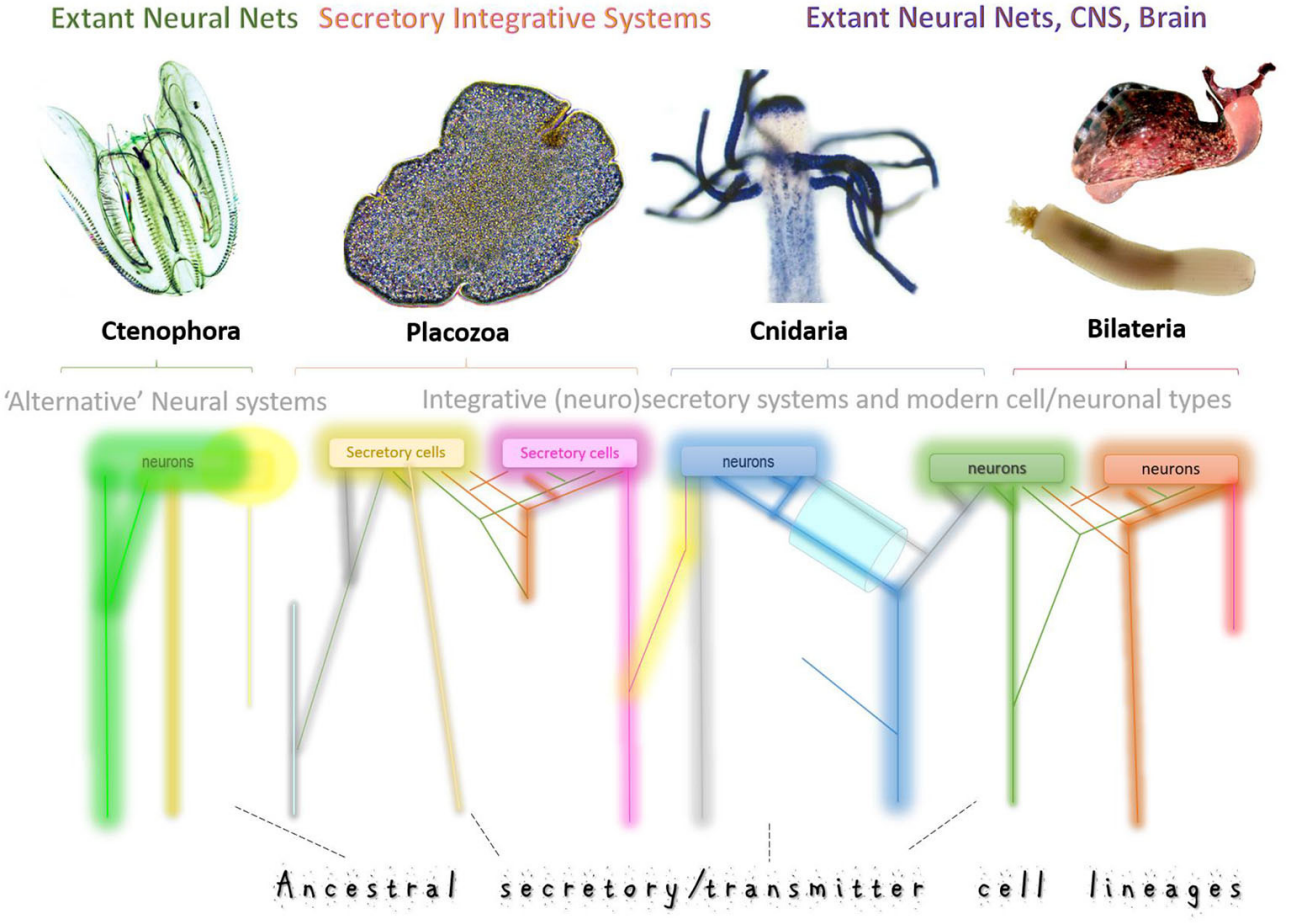
Multiple origins of neurons and secretory cells. Schematic illustration of the ancestral cell lineages (different color trajectories) that led to the exant neural systems in four basal metazoan clades with Placozoa as nerveless animals. Neural systems might consist of genetically diverged cell types with different ancestries, gene regulatory networks, and signal molecules. This diagram integrates both the hypothesis of independent origins of neurons (as in ctenophores, Moroz et al., 2014) and the sister-cell model (Arendt et al., 2016), which suggests that novel neuronal types arise in pairs, through sub-specialization of ancestral cell types. Thus, sister neuronal subtypes can share gene-regulatory networks, perhaps, evolutionary conserved developmental pathways, and are expected to have more similar expression profiles than each of them compared to other neuronal types. The key prediction of this model is that gene expression profiles from sister-cell types will form a hierarchical tree structure in phylogenetic reconstructions. A complementary model predicts that neurons and novel neuronal subtypes arise through “co-options” or “fusions” of regulatory modules and pathways “recruited” from genetically unrelated cell types. As a result, their expression profiles would be substantially different, leading to net-type rather than tree-type cellular genealogies in phylogenetic reconstructions. We expect that both scenarios can coexist in any given nervous system. But the tremendous diversity of neural systems across phyla suggests variable contributions of each historical scenario. Combining tools of (i) statistical geometry, artificial intelligence and (ii) modern phylogenomics with (iii) massive parallel single-neuron transcriptome profiling would allow us to unbiasedly reconstruct the genealogy of neurons by testing the treeness statistics as it was recently used for cancer and placental cells. The top illustrations are photos of *Mnemiopsis, Trichoplax, Podocoryne, Priapulus*, and *Aplysia*. Some cell lineages (different colors) might become eliminated in the course of evolution (loss) or be expanded or evolved in parallel from different secretory cell types.

In 1968–1974 these questions were asked from an evolutionary standpoint, and Dmitry Sakharov had proposed the hypothesis of neuronal polygeny (=multiple origins of neurons) (Sakharov, 1970a,b, 1972, 1974a,b). Using minimal comparative data available 50 years ago, Sakharov suggested that **neurons evolved from** ***genetically different*** **secretory cells**. The evolutionary view of neuronal evolution can be summarized as follows. Each of these populations of secretory cells could use chemically distinct transmitter(s) and different (distant) receptors for communications in early neural systems, where synapses *are not* required. Ancestral diversity of secretory cell types (=secretory phenotypes) has been preserved over 500+ million years of biological evolution, forming lineages of homologous neurons across phyla. Thus, neurons are different because they have different genealogies. Subsequent functional “demands” and specifications could further tune these different ancestral neurosecretory phenotypes. In other words, the traditional *one-root genealogy* of neurons was transformed into *multiple genealogies* or a net of phyletic cell/neuronal lineages, as schematically presented in Figure 1.

The polygeny hypothesis stated that the transmitter-based primordial diversity of chemical signaling networks and mechanisms is the cornerstone for any nervous system organization. It was also suggested that neurons could evolve from heterogenous secretory cells in different tissues, or embryonic layers. In other words, neurons could evolve more than once within early precursors of ectoderm, endoderm, or mesoderm and/or other tissues. The presence of many transmitters in extant nervous systems reflects the complex chemical organization or *chemical wiring* of early neural systems. Extant neural systems might preserve at least some ancestral secretory lineages (and gene regulatory networks/modules) over many millions of years.

The polygeny hypothesis also proposed the criteria and predicted neuronal homologs across taxa or within evolutionary different cell type lineages using modern terms.

Unique serotoninergic, catecholaminergic, and peptidergic homologous neurons had been identified in mollusks (Sakharov, 1974b, 1976; Weiss and Kupfermann, 1976). Thus, even in the 1970–1990s, these findings provided clear illustrative examples for **the existence of conservative neuronal cell types separated by million years of divergent evolution**. Again, gastropod mollusks were used as reference species (Sakharov, 1976; Weiss and Kupfermann, 1976; Gillette and Davis, 1977; Moroz et al., 1997; Sudlow et al., 1998). Remarkably, some homologous neuronal cell types (such as a pair of serotonergic MCC interneurons in Euthyneura) preserved their neurotransmitter identities and functions for 380 million years (Moroz, 2018). This hypothesis provided the versatile chemical foundation to reconstruct neuronal evolution where evolutionary innovations in neurosecretory and behavioral phenotypes multiply.

Below, we summarize three conceptual aspects of electrical vs. chemical signaling paradigms to understand neuronal evolution. (1) History of the idea to trace the origins of neurons from secretory cells. (2) Postulates that substantiate different evolutionary scenarios for neuronal evolution. (3) Prospectives of multi-transmitter brain organization and transmitter-dependent behaviors essential to understanding the grammar of neural systems. The elusive chemical syntax of neural ensembles can explain brain operations' emerging properties, eventually leading to basal cognition (Levin et al., 2021; Lyon et al., 2021).

## Brief History of The Idea

The diversity of low molecular weight and peptide transmitters in all animals triggered several attempts to incorporate transmitter signaling in neuronal evolution models. Several recent publications provided different historical outlines and perspectives of this endeavor (Moroz, 2009, 2014; Moroz and Kohn, 2015, 2016; Arendt, 2021; Jekely, 2021; Moroz et al., 2021), which are not yet integrated into a comprehensive and unbiased review of the subject.

### Electrical Paradigm

The sensory-effector-contractility scheme of neuronal operation was the dominant model in considering the neuronal evolution, which can be traced to the Parker's elementary neural system (Parker, 1919; Pantin, 1956; Grundfest, 1959, 1965; Passano, 1963; Horridge, 1966, 1968). Mackie (1970) outlined the elegant theory of the neuronal origin from myoepithelial-type cells (like in extant cnidarians, Mackie, 1970). Within this theoretical framework, Mackie and his colleagues further developed the concept of parallel electrical signaling systems (often coupled to contractility), supported by the widespread distribution of the epithelial conductive pathways (Anderson, 1980; Satterlie and Spencer, 1987; Mackie, 2004; Satterlie, 2015). Ancestral neuron-muscle relationships have been emphasized in these models.

### Chemical Paradigm

In 1954–1959 Clark, Haldane, and Grundfest were the first students of neuronal evolution who proposed *the origin of neurons from secretory cells* (Haldane, 1954; Clark, 1956a,b; Pantin, 1956; Grundfest, 1959). These hypotheses provided transmitter-centric prospects in deciphering neuronal evolution, in contrast to earlier concepts, which were primarily based on electrical, reflective paradigms (Kleinenberg, 1872; Claus, 1878; Hertwig and Hertwig, 1878, 1879, 1880; Chun, 1880; Hertwig, 1880; Parker, 1919; Wyman, 1925; Pantin, 1956). At that time, in the 1950–1960s, the distribution of signal molecules across phyla was mostly unknown, but neurotransmitter functions of acetylcholine, monoamines, and several neuropeptides have been established (Valenstein, 2005).

Nevertheless, the dominant view was that neurons had a single origin, and later in the evolution, the diversity of transmitters increased following the classical single Tree of Life model. Lenz specifically stressed this point in his influential work and the book (Lentz, 1966, 1968). And the neuronal monophyly model has not been challenged by other authors. For example: “The conceptual model of the ancestral neuron, considered as the phylogenetic derivative of an undifferentiated and pluripotential epithelial cell, is that of a functionally versatile structure, equally endowed for the dispatch of long-distance and localized chemical signals. The neurosecretory neuron has remained closer to the nerve cell precursor than has the conventional neuron with its specialization for synaptic transmission.” (Scharrer, 1976).

In contrast, the independent origins of neurons from multiple types of secretory cells is a more realistic reconstruction of neuronal evolution (Sakharov, 1974b; Moroz, 2009, 2014; Moroz and Kohn, 2016). The postulates derived from this hypothesis are summarized below. Electrical vs. chemical-centered hypotheses of neuronal origins are complementary. But it was stressed that **transmitters made neural systems** as integrative ensembles, where the transmitter operated as a “*versatile glue”* recruiting many proto-neurons and their effectors together to form biologically relevant behaviors (Moroz et al., 2021).

## Postulates of The Polygeny Hypothesis

1. ***In early metazoans, neurons evolved from genetically (genealogically)*** ***different secretory cells that used multiple transmitters to communicate and*** ***integrate behaviors*** ***without synapses***. From this starting point, the evolutionary innovations multiply. Nerveless Placozoa and Porifera are two animal lineages that preserved such ancestral intercellular communications and, likely, the integration of behavior in a “pure” non-synaptic form (i.e., without any recognized electrical or chemical synapses, Moroz et al., 2021). In other words, both **the diversity of transmitters and their receptors** ***predated* the origins of neural systems**. The recruitments of classical transmitters and (neuro)peptides in early developmental control (Buznikov, 1990) might reflect this ancestral pre-neuronal integrative functions of these intercellular signal molecules (Koshtoyants et al., 1961; Buznikov et al., 1964, 1968, 1970, 2001, 2005, 2010; Buznikov and Shmukler, 1981; Buznikov, 1991; Shmukier and Buznikov, 1998; Levin et al., 2006).
2. In evolutionary terms, **a neuron is a functional (not genetic) category**. The genetic category is referred to the scenario that all neurons are derived from the *same* ancestral cell lineage. Therefore, all neural systems and neuronal cell types are homologous because of their shared *genetic* ancestry. Alternatively, the functional category signifies examples of convergent evolution when similar chemical and physiological constraints resulted in similar neuronal phenotypes. This view does not prevent establishing evolutionary lineages of homologous cell types within particular taxonomical units such as classes, subclasses, orders, families, and genera. The cell-lineage-specific homologies across phyla are still a challenge (Tarashansky et al., 2020).
3. *What is a neuron?* As their “ancestors,” all modern **neurons are polarized secretory cells specialized for directional active conducting and release of more than one transmitter**: usually 2–5 peptides and a low-molecular transmitter(s) (Weiss et al., 1992; Moroz et al., 2005, 2006; Moroz and Kohn, 2010; Cropper et al., 2018; Merighi, 2018; Nassel, 2018; Svensson et al., 2018). These features enable neurons to convey signals, primarily chemical, beyond their immediate neighbors and without affecting all intervening cells *en route*. Evolutionary elaborated memory capabilities of neurons are essential to generate stereotyped and learned behaviors within the same cell ensembles (Kandel, 2001; Walters and Moroz, 2009; Walters and Williams, 2019).
4. **An ancestral mode of intercellular communication mediated by early neurons was a non-synaptic transmission** (= volume or paracrine secretion; Moroz, 2009, 2014). The early directional signaling was achieved due to the differential cell-specific expression of receptors for secreted signaling molecules and diffusion/microanatomical constraints.
5. As neurons, **synapses evolved independently in animal lineages** and later in evolution (Moroz and Kohn, 2016). Early neural systems were without synapses but with dozens and even hundreds of signal molecules (=small transmitters and secretory peptides) and multiplicity of their receptors (Moroz et al., 2021). These classes of signal molecules formed the chemical and dynamic connectome or a sort of multi-transmitter “*glue”* uniting neurons to generate stereotyped and learned behaviors.
6. **Early neurons were primarily genetically different because of their genealogy**. The first level of evolutionary constraints can be traced back to deep ancestry of complex life cycles of eukaryotes and the nerveless ancestor of all animals (=Urmetazoan). Alexey Zakhvatkin (1906–1950) originally proposed this hypothesis (https://www.si.edu/object/siris_sil_363532), which obtained additional evidence (Mikhailov et al., 2009; Tikhonenkov et al., 2020). The subsequent functional specification within distinct cellular lineages results from parallel evolutionary processes, perhaps similar to the cell-sister type hypothesis (Arendt, 2008; Arendt et al., 2016), and Figure 1.
  Of note, even unicellular eukaryotes have many cell types because of their complex life cycles. Cell types in a given unicellular eukaryote are separated in time of development, including the formation of colonial organisms. In evolution of the lineage that led to animals' multicellularity, the preexisting *temporal separation* of cell types was switched to the *spatial co-existence* of similar ancestral cell types (Mikhailov et al., 2009; Tikhonenkov et al., 2020). The Urmetazoan could possess 10–50 distinct cell types (Moroz, 2018; Sebe-Pedros et al., 2018; Musser et al., 2019). Some of these early cell types could be traced back to the complex life cycles of unicellular and colonial eukaryotes.
7. **Every neural system is chemically and genetically chimeric**. This prediction is the most straightforward consequence of the neuronal polygeny hypothesis. Some ancestral neural lineages were lost in evolution, but the core genomic regulatory modules (transcription factors, enhancers, etc.) were preserved in extant nervous systems as decedents of early cell types. Most invertebrate ganglia, neural “circuits” or neural ensembles are composed of different cell lineages with distinct secretory phenotypes and evolutionary histories. I predict the reconstruction of hundreds of genealogies for metazoan secretory cells and neurons in particular. Neurons might evolve from ectodermal, entodermal, and mesodermal-type derivatives. See illustrative examples from the sea urchin (Wei et al., 2011), cnidarians (Nakanishi et al., 2012), including recent scRNA-seq work (Arendt, 2019; Siebert et al., 2019), and ctenophores (Moroz et al., 2014; Moroz, 2015a). Trans-differentiation with transmitter phenotype switching both in development and adult brains is possible (Spitzer, 2017; Bertuzzi et al., 2018; Meng et al., 2018; Ferrarelli, 2020; Li et al., 2020). But it might be a relatively rare event stressing both modularity and substantial evolutionary conservation of secretory specificity within the lineages of homologous neurons.
8. By acting within synaptic clefts and beyond, the transmitters are **multi-level integrators of behaviors and behavioral choice**. *Transmitters could be versatile integrative factors* that non-synaptically unite different effectors (ciliated, secretory, contractile, immune cells, etc.) in early animals. As a result, the tightly coupled integrative *transmitter systems (secretory phenotypes) are evolutionary conservative*. Thus, the transmitter specificity can be instrumental in deciphering the *homologous behaviors* (=transmitter-induced motor outputs and behaviors). For example, serotonin acts as the integrator of behavioral [feeding] arousal in annelids (Lent, 1974, 1984, 1985; Lent et al., 1991) and mollusks (Kabotyanskii and Sakharov, 1991; Moroz, 1991; Gillette et al., 2000) and many other bilaterians (e.g., Sakharov, 1990). Serotonin has one of the most evolutionary conservative systemic functions across bilaterians. Dopamine and other catecholamines also integrate behaviors in various evolutionary lineages (e.g., Livingstone et al., 1980; Kravitz, 1988; Moroz, 1991), but the systemic functions of dopamine are less evolutionary conserved than those for serotonin. These functional differences might be related to the different chemical reactivity and stability of two transmitter molecules. ***Serotonin is an antioxidant*** capable of terminating free radical oxidative reactions (therefore, be more “resistant” to bioenergetic perturbations and more evolutionary stable). In contrast, dopamine is easily oxidized with several potentially toxic products (often leading to neurodegeneration).
9. With **more than 20 small and 100+** **peptide transmitters in nearly every nervous system**, their chemical balances provide unprecedented opportunities for evolutionary innovations, behavioral controls, and behavioral choice—all uniquely realized in different animal groups. Thus, “**transmitters made nervous system**” (Moroz et al., 2021). The foundation of brain languages is the *multi-transmitter organization of early neural systems*. Thus, ***both*** low molecular weight transmitters (amino acids such as glutamate, aspartate, glycine, as well as ATP, NO, protons) and short peptides were the first transmitters or co-transmitters. Multi-transmitter chemical wiring and integration were imperative both in Precambrian metazoans and the present-day animals. Some parasitic groups might have a reduced set of their neurotransmitters because of secondary simplification (e.g., orthonectids with about two dozen neurons in the entire CNS; Slyusarev and Starunov, 2016; Slyusarev et al., 2020).
10. **The ancestral non-synaptic transmission has not disappeared in the course of evolution**, contributing to the neuronal integration in extant neural systems. Paracrine, non-synaptic communication is also known as the volume transmission (Agnati et al., 1995, 2006, 2010; Zoli et al., 1998; Nieuwenhuys, 2000; Ridet and Privat, 2000; Sykova, 2004; Trueta and De-Miguel, 2012; Taber and Hurley, 2014; Noble et al., 2018). Even non-synaptic organization of central pattern generators is theoretically possible; it can be illustrated by mathematical modeling of chemical gradients and generation of rhythmic behaviors without synapses. Changeable chemical gradients and oscillations of extra-synaptic neurotransmitters have also been experimentally detected *in vivo* using physically isolated neurons as a biosensors (Chistopol'skii and Sakharov, 2008; Chistopolsky et al., 2008; Dyakonova et al., 2015).

Ctenophores or comb jellies seem to present the most extreme case of multiple origins of neurons and synapses (Moroz et al., 2014; Moroz and Kohn, 2016), with the remarkably different multi-transmitter set. In this early-branching animal lineage (Whelan et al., 2017), there are two morphologically functional, molecularly and, perhaps, genetically different neural systems: (i) skin nerve net and (ii) even more diffused cells in the mesoglea. The mesogleal neuroid elements share their phenotypes with muscle cells (Norekian and Moroz, 2019a,b, 2020). This situation might be a relict; does it reflect the origins of some populations of neurons and muscles from the same evolutionary predecessors? A similar situation might be in cnidarians as outlined in the hypothesis of G. Mackie (1970). However, in this scenario, the evolutionary predecessors of neurons and muscles were myoepithelial cells. The emerging single-cell sequencing data in *Hydra* (Siebert et al., 2019) showed that selected muscle and neuronal cells in cnidaria might share some transcriptional factors summarized in the recent review (Arendt, 2021). Of note, striated muscle cells also evolved at least two-three times in evolution (Steinmetz et al., 2012).

## Questions and Prospective for Exprerimental Validation

The polygenesis hypothesis, and many of its predictions related to reconstructions of cellular genetic relationships, can be tested using single-cell “omics” approaches. The initial data from different phyla and observed unprecedented diversity of molecular phenotypes (Sebe-Pedros et al., 2018; Cocanougher et al., 2019; Musser et al., 2019; Siebert et al., 2019) seems to favor the hypothesis of multiple origins of neurons and the existence of numerous cell-type-specific phyletic lineages. However, the challenge is integrating vast comparative data (with expected hundreds of cell-type-specific lineages across thousands of species) with real-time physiology of individual cells and their ensembles in each representative species. It might take decades, but a new evolutionary theory for neural diversity and functions needs interdisciplinary studies. I envision a Periodic System of Cell Types—the natural genealogical classification of cell phenotypes and states integrated with evolutionary cell trees of Life and *predictive power*. It can be a conceptual analog to the Periodic System of Chemical Elements; that is, the position of an element in the Periodic System predicts its properties (e.g., inert gases or metals). Similarly, the ideal classification of cell types can predict their functional features and constraints (Moroz, 2018). The fundamental questions to be addressed can be broadly divided into two overlapping long-term objectives: (i) deciphering neural evolution vs. (ii) decoding chemical networks for intercellular communications, including methodology to reveal numerous chemoconnectomes unbiasedly.

### Deciphering Neuronal Evolution

Single-cell comparative data and novel informatics theory are needed for multiple cross phyla genealogies. But, no criteria for cell-specific homologies across phyla have been established and experimentally validated. Some approaches are suggested (Tarashansky et al., 2020), but true homology can only be found with multiple cross-validated criteria, including identifying potential continuity of homologs tracing intermedial species. Here, the lack of required comparative data from “minor” phyla and classes is a significant bottleneck. There are ~35 metazoan phyla and 100+ classes with dozens of eukaryotic lineages related to metazoans. The most critical basal metazoan groups to be investigated are Placozoa, Porifera, and Ctenophores. For representatives of these groups and other reference species (Striedter et al., 2014), the following questions need to be addressed.

*Are there yet unknown transmitters?* Prediction: it can be dozens of novel small (neuro)transmitters and many thousands of novel neuropeptides. The secretory organelles are highly conserved across eukaryotes. The recent comparative study on the choanoflagellates (the sister group to Metazoa) clearly illustrated a polarized localization of putative but quite diverse secretory vesicles in two model species *Salpingoeca rosetta* and *Monosiga brevicollis* (Gohde et al., 2021). However, it is unclear how many signal molecules can be co-released? What are the functions of such paracrine secretion in choanoflagellates?

*What is the contribution of synaptic vs. non-synaptic release across different animal lineages?* For most transmitters, we anticipate a broad spectrum of variations. A synapse can be at one part of the spectrum, with the highly localized transmission within the synaptic cleft constraints, to “true” hormonal distance signaling. Volume transmission is not only restricted by diffusion rates of signal molecules. Tissue micromechanics, cilia-induced vortexes, and dynamic extracellular space can also clearly increase passive diffusion rates in unicellular and colonial organisms and multicellular, primarily nerveless, animals such as sponges and placozoans.

*How many types of synapses, and what is their natural/evolutionary classification?* Synapses in ctenophores and cnidaria are poorly analyzed. Nothing is known about volume transmitters in these organisms as in the majority of bilaterians. Volume transmission in nerveless animals such as placozoans and sponges has not been quantitatively measured.

*Do transmitters evolve? Are there any constraints and trends in the evolutionary selection of (neuro)transmitters and synapses?* Both functional (e.g., chemical stability vs. reactivity [antioxidant/prooxidant properties], synthesis, inactivation, etc.) and evolutionary constraints (pre-adaptations, ecology, and lifestyles) have to be considered. The chemical/secretory organization and relationships among the digestive, immune and neural cell types are unknown for basal metazoans.

### Deciphering Chemoconnectomes and Chemical Syntaxis of Neural Systems

A *chemoconnectome* is defined as an entire set of neurotransmitters, neuromodulators, neuropeptides, and receptors supporting chemical transmission in an animal (as illustrated for *Drosophila* by Deng et al., 2019). However, visualization of dynamic chemoconnectomes (which can change in time: from milliseconds to hours and days), is a much greater challenge than reconstructing traditional connectomes, static descriptions of synaptic wiring. This challenge demands conceptually new and innovative methods and theories to simultaneously image dozens of specific molecules over broad ranges of transmitter concentrations (nanomoles to micromoles) in real-time. Unfortunately, most current bioanalytical approaches measure one or a few neurotransmitters at a time, and only for narrow concentration ranges. The 4D dynamic (3D space+time) of complex (extracellular) *milieus inferiors* with hundreds of signal molecules (spread from nanoliter to milliliter volumes) is the Frontier in cell, developmental, and evolutionary biology as well as biomedicine.

Multiplexed nanotools (Farsi et al., 2016; Jing et al., 2018; Wu et al., 2018; Dinarvand et al., 2019; Zeng et al., 2020) are instrumental in visualizing cell-specific secretion of co-transmitters and the actual balance of neurotransmitters as stereotyped and learned behaviors are generated.

The combinatorial power of chemical interactions is enormous, but constraints of neurotransmitters signaling also exist, observed in well-defined phenomena of transmitter-dependent behaviors (Dyakonova and Sakharov, 2019). Methodological and theoretical efforts would decipher still elusive “neuronal syntax” (Buzsaki, 2010) of the electrochemical brain grammar (as “words,” “sentences,” or other hierarchically organized “quanta” of bio-information), which, I think, is the primary chemical, and transmitter-based, in its nature.

I would conclude that the current biodiversity of species with the astonishing diversity of secretory and signaling mechanisms, neurons and synapses, neural and alternative integrative systems are true *Gifts of Nature* to neuroscientists and humankind. We are only getting the first surprises from these gifts. We are only starting to taste novel fundamental insights and paradigm shifts in this endeavor. It might be controversial, but the shortcut to better understanding our brains and neurological disorders and regenerative medicine of the future is studying small creatures in the world ocean. Admittedly, not all marine creatures can be brought to the lab and cultured. But we now have the capacity to bring labs to the sea (Moroz, 2015b) and expand frontiers of the living world and ourselves.

## Author Contributions

The author confirms being the sole contributor of this work and has approved it for publication.

## Conflict of Interest

The author declares that the research was conducted in the absence of any commercial or financial relationships that could be construed as a potential conflict of interest.
